# Screening Five Qi-Tonifying Herbs on M2 Phenotype Macrophages

**DOI:** 10.1155/2019/9549315

**Published:** 2019-01-15

**Authors:** Yi-Xin Jiang, Yan Chen, Yue Yang, Xiao-Xia Chen, Dan-Dan Zhang

**Affiliations:** ^1^Institute of Interdisciplinary Integrative Medicine Research, Shanghai University of Traditional Chinese Medicine, Cailun Road 1200, Shanghai 201203, China; ^2^School of Pharmacy, Shanghai University of Traditional Chinese Medicine, Cailun Road 1200, Shanghai 201203, China

## Abstract

Tumor-associated macrophages (TAMs) with M2 phenotype play an essential role in tumor microenvironment (TME) during the progression and development of numerous cancers and associated with poor prognosis. Thus, regulation of TAMs polarization emerged as a new strategy for tumor immune therapy. According to Traditional Chinese Medicine (TCM) theory, herbs with Qi-tonifying character are involved in improving the defense capacity of immune system. In this study, we screened extracts and ingredients from five Qi-tonifying herbs exhibiting an inhibitory effect on M2 polarization of murine macrophages RAW264.7 induced by IL-4 and IL-13. Among these candidates, total flavonoids from Glycyrrhiza Radix et Rhizoma (TFRG) and ethanol extract of Ginseng Radix et Rhizoma significantly inhibited the expression of Arginase-1 (Arg-1) (above 90% at 100*μ*g/mL), one of the phenotype markers of M2 macrophages. The inhibition of total saponins of Ginseng Radix et Rhizoma, ethanol extract of Cordyceps, ethanol extract of Acanthopanacis senticosi Radix et Rhizoma Seu caulis, and ethanol extract of Astragali Radix reached above 50% at 100*μ*g/mL. The inhibition of ingredients including glabridin, isoliquiritin apioside, lysionotin, cordycepin, astragaloside IV, and calycosin reached above 50% at 50*μ*M. Then, we investigated the molecular mechanisms of TFRG. TFRG abolished the migration of murine breast cancer 4T1 stimulated by the conditioned medium from M2 macrophages (M2-CM). In addition to Arg-1, TFRG also antagonized the IL-4/13-mediated mRNA upregulation of the M2 markers including found in inflammatory zone 1 (FIZZ1), chitinase-3-like protein 3 (YM1), and mannose receptor (CD206) and upregulated the expression of inducible nitric oxide synthase (iNOS), one of the M1 markers. The further exploration showed that TFRG decreased the phosphorylation of STAT6 and increased the expression of miR-155. Our study provides a series of potential immune regulating natural products from five Qi-tonifying herbs on M2 phenotype. For instance, TFRG suppressed M2 polarization of macrophages partly by inactivating STAT6 pathway and enhanced the level of miR-155 to regulate the expressions of M1 and M2 markers.

## 1. Introduction

Macrophages are essential immune cells in protecting human body from external threats such as bacterial and virus threats, while their functions switch to associate tumor development under tumor microenvironment as tumor associated macrophages (TAMs). TAMs are involved in micrometastasis niches and immune escape [1]. The population of TAMs is negatively correlated with clinical prognosis in many kinds of malignant tumors. So TAMs are considered as a new therapeutic target in the field of tumor immune therapy [2].

Macrophage polarization is two extremes of situations after mononuclear cell activation, and various microenvironment signals induce its polarization. According to different functional characteristics and its induced consequence, macrophages are usually divided into M1 and M2 [3]. Classically activated macrophages (M1) are activated by T helper (Th)-1 cytokines such as interferon gamma (IFN-*γ*) and bacterial wall components lipopolysaccharide (LPS). These macrophages have antitumor effect due to the cell toxicity of nitric oxide induced by inducible nitric oxide synthase (iNOS). Alternatively activated macrophages (M2) are activated with T helper (Th)-2 cytokines such as IL-4 and/or IL-13[4], which have opposite function to that of M1.

Arginase-1 (Arg-1) presents in both murine and human with M2 induction while being very low in M1 phenotype; that indicates that it is an optimal specific marker of M2. Inhibitors of Arg-1 maybe have effect on regulation immune microenvironment targeting TAMs. The expression of Arg-1 requires tight control at transcriptional level. There are several pivotal transcription factors that regulate the expression of Arg-1 such as Signal Transducers and Activators of Transcription 6 (STAT6). The expression of Arg-1 strictly depends on STAT6 in the presence of IL-4 and/or IL-13 during M2 activation [5]. Inhibition of STAT6 in TAMs attenuates tumor growth and metastasis [6].

Numerous recent studies reveal that some microRNAs (miRNAs) are involved in macrophage polarization by regulating transcription factors [7]. Among these miRNAs, miR-155 targeted IL-13R*α*1 via STAT6 signal pathway to suppress M2 phenotype of macrophages [8].

According to TCM, constant motion and transformation of Qi maintain body internal balance. And the occurrence of tumor is caused by the deficiency of Qi. One of the key principles to cancer intervention guided by TCM is strengthening Qi. Qi can improve the immune system against pathogenic factors. Qi-tonifying herbs or their formulas are usually used to prevent cancer recurrence and metastasis of postoperative cancer patients in China [9].

So, we selected five Qi-tonifying herbs with high frequency in clinical practice against cancers and screened their effects on M2 macrophages.

## 2. Materials and Methods

### 2.1. Drugs and Reagents

Glycyrrhiza Radix et Rhizoma (Gancao, RC), Ginseng Radix et Rhizoma (Renshen, RS), Cordyceps (Dongchongxiacao, DCXC), Acanthopanacis senticosi Radix et Rhizoma Seu caulis (Ciwujia, CWJ), and Astragali Radix (Huangqi, HQ) were purchased from Yanghetang Pharmaceutical Company (Shanghai, China). Botanical identification of these herbs was performed by Shanghai Institute for Food and Drug Control (SIFDC). Ingredients (Table 1) were purchased from Shanghai Tauto Biotechnology Company with purity more than 98% by HPLC. Mouse IL-4 and IL-13 were purchased from Peprotech (Rockville, MD, USA). Fetal bovine serum (FBS) was purchased from Hyclone (Waltham, MA, USA). Thiazoyl 3-(4,5-Dimethyl-2-thiazolyl)-2,5-diphenyl-2H-tetrazolium bromide (MTT) and dimethylsulfoxide (DMSO) were purchased from Sigma (St. Louis, MO, USA). Trizol, High-Capacity cDNA Reverse Transcription Kit, Real Master Mix SYBY Green, Prestained protein Marker, and BCA protein concentration assay kit were purchased from Invitrogen (Carlsbad, CA, USA). TaqMan MicroRNA primers, transcription kit, and universal PCR master mix were obtained from Applied Biosystems (Foster City, CA, USA). PVDF membranes and ECL kit were purchased from Millipore (Bedford, MA, USA). Arg-1, iNOS antibody, and secondary antibody were purchased from Abcam (Cambridge, UK). Antibody phosphor-STAT6 and STAT6 polyclonal antibodies were purchased from Cell Signaling Technology (Beverly, MA, USA). RIPA Lysate was purchased from Beyotime Biotechnology Company (Haimen, China). Primers were synthesized by Shanghai Bioengineering Company. Other reagents were analytical grade.

### 2.2. Preparation of Extracts from Five Chinese Herbs

To prepare ethanol extracts, 100 g of each dried herbs was sliced and extracted with 1 L of 70% ethanol at 80°C for three times. Obtained ethanol extracts were evaporated under reduced pressure at temperature 60°C and stored at -80°C. Extracts were dissolved with DMSO before use. TFRG was obtained as described previously [10], and no endotoxin existed in it by endotoxin test.

### 2.3. Cell Culture

Mouse macrophage cell line RAW264.7 and mouse breast cancer cell line 4T1 were obtained from ATCC. RAW264.7 cell and breast cancer 4T1 cell were cultured in RPMI-1640 medium which contained 10% FBS, placed in saturated humidity, 5% CO_2_ incubator, and incubated at 37°C constant temperature. Take 10 to 15 generations of cells for these experiments.

### 2.4. Preparation of Conditioned Medium

RAW264.7 cells were seeded in 30mm dish and cultured overnight. Then cells were treated with/no different concentrations (25, 50*μ*g/mL) of TFRG and IL-4 (20*μ*g/mL)/IL-13 (20*μ*g/mL) induction for 24 h in serum free medium. The culture supernatants (conditioned medium, CM) were collected and centrifuged to remove cellular components and stored in 4°C before use.

### 2.5. Cell Viability Assay

Cell viability was measured using MTT assay [10].

### 2.6. Trans-Well Migration Assay

Cell migration assay was performed using the Trans-well system (24-wells, 8-*μ*m pore size, Corning Costar, Lowell, MA, USA). Briefly, 4T1 cells were harvested and suspended in serum-free medium; then 5 × 10^4^ cells were added to the upper wells. 600*μ*L conditioned medium from each group was added to the lower chamber. After treatment for 24 h, the cells attached to the lower surface were fixed with methanol and stained with 0.1% crystal violet. The number of cells that migrated was counted in five randomized fields using an Olympus light microscope.

### 2.7. qRT-PCR

Total RNA was extracted from cells using TRIzol regents according to the manufacturer's protocol. The sequences of these primers are as shown in Table 2. The level of GAPDH mRNA was used as an internal standard and Applied Biosystems 7500HT Fast Real-Time PCR System was used to perform the reactions. PCR reaction conditions were the following: 95°C for 10 minutes, 50°C for 2 minutes, 95°C for 15s, 60°C for 60s, and 40 cycles.

### 2.8. TaqMan® MicroRNA Real-Time RT-PCR Assays

Mmu-miR-155 and the housekeeping gene U6 were carried out according to the manufacturer's protocol [11]. These samples were measured in triplicate cases. U6 endogenous control was used for normalization, and expression levels were presented as 2^−ΔΔCT^ with standard deviation.

### 2.9. Western Blot Analysis

Cell extracts were collected using RIPA buffer and separated on 4−12% SDS-PAGE gels followed by the transfer to PVDF membrane. The membranes were blocked with 5% skim milk and incubated with the appropriate primary antibodies. Antibodies used include Arg-1, iNOS, phosphor-STAT6, STAT6, and GAPDH.

### 2.10. Statistical Analysis

Data were reported as the mean ± standard deviation for each individual experiment. Statistical analysis was performed using Student's* t*-test.* P* < 0.05 was considered as significant.

## 3. Results

### 3.1. Effects of Herb Extracts and Ingredients on Arg-1 mRNA Expression and Cell Growth

These extracts and ingredients were evaluated for their effects on Arg-1 mRNA expression in IL-4/IL-13-induced murine RAW264.7 macrophages and cell growth on unstimulated macrophages with the aid of qRT-PCR and MTT.

A wide range of inhibitions in Arg-1 mRNA expression was observed with these candidates with no cell cytotoxicity (Table 3). TFRG and ethanol extract of RS showed the strong inhibition of Arg-1 mRNA expression (above 90% at 100*μ*g/mL). The inhibition of total saponins of RS, ethanol extract of DCXC, ethanol extract of CWJ, and ethanol extract of HQ reached above 50% at 100*μ*g/mL. The inhibition of ingredients including glabridin, isoliquiritin apioside, lysionotin, cordycepin, astragaloside IV, and calycosin reached above 50% at 50*μ*M.

### 3.2. TFRG Abolished Migration of 4T1 Induced by M2CM

**Figure 1:**
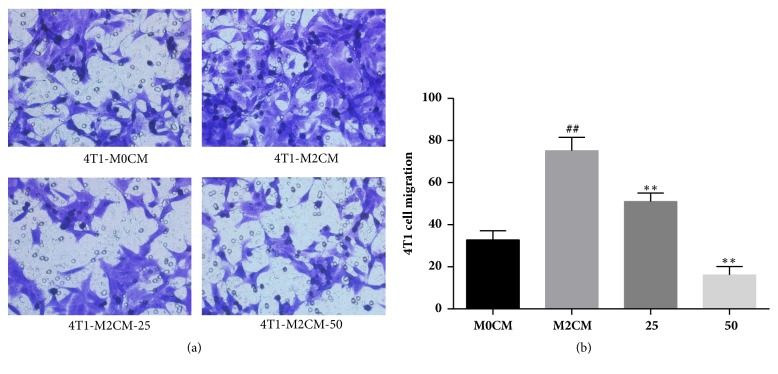
**TFRG abolished migration of 4T1 induced by M2CM**. RAW264.7 macrophages were incubated with treatment of 25 and 50*μ*g/mL TFRG and stimulated with IL-4 (20ng/mL)/IL-13(20ng/mL) for 24h. Un-treatment macrophages are termed as M0 group. The conditioned medium of each group was collected and put in the lower chamber of trans-well, and 4T1 cells (5*∗*10^4^ cells/well) were put in the upper wells of trans-well to migrate for 24h. Values are presented as the mean ± standard deviation from three replicates. ^##^*P* < 0.01 versus M0CM group; ^*∗∗*^*P* < 0.01 versus M2CM group.

Since TFRG showed the strongest inhibition of Arg-1 mRNA among these extracts without cytotoxicity, we wondered that whether TFRG can influence the function of M2 macrophage. Accumulated evidence suggests that M2 macrophages assist cancer cell to metastasis and worsen TME [12]. The conditioned medium from M2 macrophages after IL-4/IL-13 induction (M2CM) significantly improved the migration ability of breast cancer 4T1 cells* in vitro*. The conditioned medium from TFRG pretreatment M2 macrophages reduced the increased migration ability of 4T1 cells in a dose-dependent manner (Figure 1).

### 3.3. TFRG Suppressed Arg-1 Expression at mRNA and Protein Level

**Figure 2:**
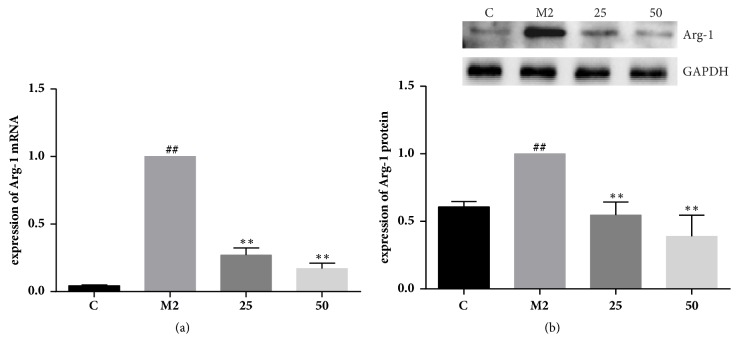
**Effect of TFRG on Arg-1 expression at gene and protein level in IL-4/IL-13-induced murine RAW264.7 cells**. (a) Effect of TFRG on Arg-1 mRNA in IL-4/IL-13-induced murine RAW264.7 cells. RAW264.7 macrophages were incubated with pretreatment of 25 and 50*μ*g/mL TFRG and stimulated with IL-4 (20ng/mL)/IL-13 (20ng/mL) for 24h. Values are presented as the mean ± standard deviation from three replicates. ^##^*P* < 0.01 versus C group; ^*∗∗*^*P* < 0.01 versus M2 group. (b) Effect of TFRG on Arg-1 protein in IL-4/IL-13-induced murine RAW264.7 cells. RAW264.7 macrophages were incubated with pretreatment of 25 and 50*μ*g/mL TFRG and stimulated with IL-4 (20ng/mL)/IL-13 (20ng/mL) for 24h. Values are presented as the mean ± standard deviation from three replicates. ^##^*P* < 0.01 versus C group; ^*∗∗*^*P* < 0.01 versus M2 group.

Arg-1, a marker for the M2 subset macrophages, hydrolyzes L-arginine into urea and ornithine and inhibits nitric oxide-mediated pathways by competing with iNOS for the same substrate L-arginine [13]. In resting macrophages, low expression of Arg-1 was detected, and the expression of Arg-1 was significantly increased after IL-4/IL-13 induction towards M2 phenotype. Thus, we investigated the possibility that TFRG blocked Arg-1 expression. To test this possibility, we determined the effect of TFRG on Arg-1 expression in induced RAW264.7 cells. Results from qRT-PCR and western blotting revealed that TFRG downregulated Arg-1 expression in a dose-dependent manner at levels of gene and protein (Figure 2).

### 3.4. TFRG Downregulated M2 Related Markers and Upregulated M1 Marker iNOS

**Figure 3:**
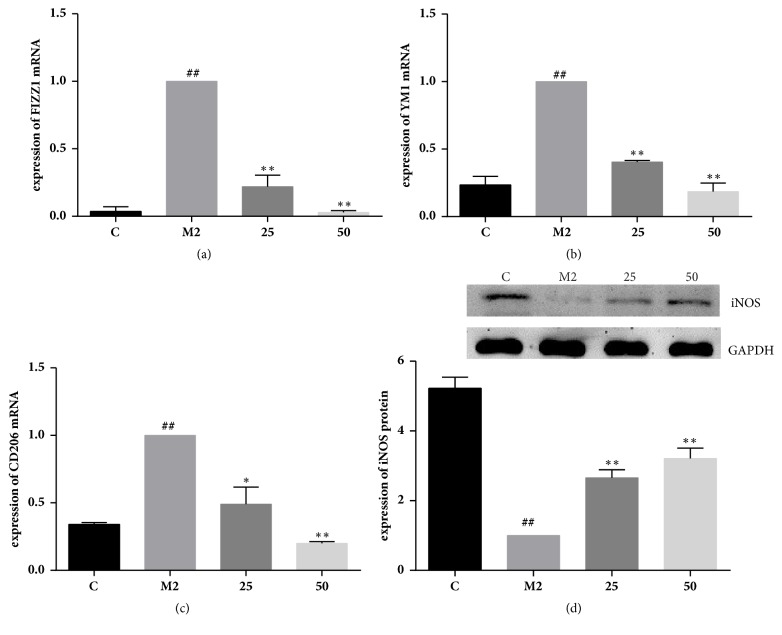
**Effect of TFRG on expression of M2-induced genes and M1 marker iNOS in IL-4/IL-13-induced murine RAW264.7 cells**. Effect of TFRG on FIZZ1 (a), YM1 (b), and CD206 (c) mRNA in IL-4/IL-13-induced murine RAW264.7 cells. RAW264.7 macrophages were incubated with pretreatment of 25 and 50*μ*g/mL TFRG and stimulated with IL-4 (20ng/mL)/IL-13 (20ng/mL) for 24h. Values are presented as the mean ± standard deviation from three replicates. ^##^*P* < 0.01 versus C group; ^*∗*^*P* < 0.05 versus M2 group; ^*∗∗*^*P* < 0.01 versus M2 group. (d) Effect of TFRG on iNOS protein in IL-4/IL-13-induced murine RAW264.7 cells. RAW264.7 macrophages were incubated with pretreatment of 25 and 50*μ*g/mL TFRG and stimulated with IL-4 (20ng/mL)/IL-13 (20ng/mL) for 24h. Values are presented as the mean ± standard deviation from three replicates. ^##^*P *< 0.01 versus C group; ^*∗∗*^*P*<0.01 versus M2 group.

Since TFRG is sufficient to reduce the expression of Arg-1 in M2 macrophages, we wondered whether the expression of other M2 markers can be also reduced by TFRG. We found that TFRG downregulated the M2 markers FIZZ1, YM1, and CD206 in IL-4/13 derived M2 macrophages (Figures 3(a)–3(c)). In addition, the M1 marker iNOS was impaired in macrophages towards M2, and TFRG restored the production of iNOS under the induction of M2 (Figure 3(d)).

### 3.5. TFRG Blocked IL-4/IL-13-Induced STAT6 Activation

**Figure 4:**
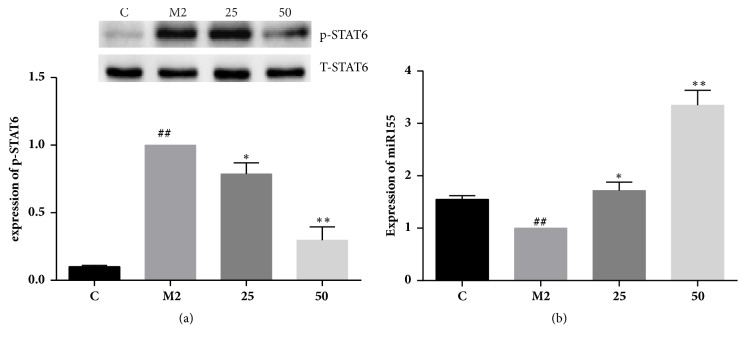
**Effect of TFRG on phosphorylation of STAT6 and miR-155 level in IL-4/IL-13-induced murine RAW264.7 cells**. (a) Effect of TFRG on phosphorylation of STAT6 in IL-4/IL-13-induced murine RAW264.7 cells. RAW264.7 macrophages were incubated with pretreatment of 25 and 50*μ*g/ml TFRG and stimulated with IL-4 (20ng/mL)/IL-13 (20ng/mL) for 30min. Values are presented as the mean ± standard deviation from three replicates. ^##^*P* < 0.01 versus C group; ^*∗*^*P* < 0.05 versus M2 group; ^*∗∗*^*P* < 0.01 versus M2 group. (b) Effect of TFRG on miR-155 level in IL-4/IL-13-induced murine RAW264.7 cells. RAW264.7 macrophages were incubated with pretreatment of 25 and 50 *μ*g/ml TFRG and stimulated with IL-4 (20ng/mL)/IL-13 (20ng/mL) for 24 h. Values are presented as the mean ± standard deviation from three replicates. ^##^*P* < 0.01 versus C group; ^*∗*^*P* < 0.05 versus M2 group; ^*∗∗*^*P* < 0.01 versus M2 group.

STAT6 signaling pathway is associated with the regulation of M2 polarization in macrophages induced by cytokines IL-4 and IL-13 [14]. To explore potential relationship between TFRG and STAT6 signaling pathway, we determined the effect of TFRG on the phosphorylation status of STAT6 in IL-4/IL-13-induced RAW264.7 cells. Western blotting showed that the level of phosphorylated STAT6 was much higher in induced M2 cells in comparison with unstimulated cells. Treatment of TFRG dependently decreased the level of phosphorylated STAT6 (Figure 4(a)). The result indicated that TFRG might exert its inhibition of M2 macrophages by the interference with STAT6 signaling pathway.

### 3.6. TFRG Induced miR-155 Expression in M2 Macrophages

Recent evidence has demonstrated that miR-155 is involved in M2 polarization [15]. To determine the effect of TFRG on miR-155 under the induction of IL-4/IL-13 in RAW264.7 cells, we pretreated cells with TFRG prior to induction. qRT-PCR showed that IL-4/IL-13 led to the decrease of the expression of miR-155 comparing with unstimulated cells. TFRG dramatically increased the level of miR-155 in RAW264.7 cells challenged by IL-4/13. It suggested that TFRG may prevent M2 polarization via miR-155 (Figure 4(b)).

## 4. Discussion

Despite the effectiveness of conventional therapies that include surgery, radiotherapy, and chemotherapy that focus on tumor cells, the survival time of cancer patients is still unsatisfying after clinical treatment. TME and its components play a critical role in recurrence and metastasis [16]. Considering the essential role of macrophages in the progression of tumor, an increasing attention has been exerted on regulating their polarization to help better clinical diagnosis, prognosis, and therapeutic applications [17]. Natural products from TCM are always evaluated on proliferation of tumor cells, and further efforts should be made to evaluate their regulation on immune cells such as TAMs.

TCM theory was developed during thousand years of clinical experience fighting against diseases, and the key principle of TCM cancer treatment is to strengthen Qi. Herbs with Qi-tonifying characteristic such as RS, DCXC, and HQ have been used with high frequency to ameliorate symptoms of insufficiency and reduction of Qi in cancer patients during tumor development or chemotherapeutic drugs administration [18]. However, Qi-tonifying herbs usually have no or less cell cytotoxicity to cancer cells. Thus, we hypothesize that Qi-tonifying herbs may have effect on M2 phenotype regulation to improve TME.

Arg-1 is well established as a major marker of M2 macrophages. Tumors of Arg-1-deficient mice were about half the size comparing with those of wild-type mice, suggesting that Arg-1 in TAMs has an essential role in tumor growth by producing polyamines to promote cell proliferation [19].

In this present study, taking advantage of the M2 phenotype cell model induced by IL-4/IL-13 on macrophage RAW264.7, we evaluated extracts and ingredients from five Qi-tonifying herbs for their inhibitory effects on IL-4/IL-13-induced Arg-1 expression. We revealed that 6 extracts and 6 compounds markedly inhibited polarization of macrophages toward M2 phenotype via inhibiting Arg-1 mRNA.

Among them, TFRG showed the strongest inhibitory effect on Arg-1 mRNA. The mechanisms of TFRG in regulating M2 polarization events need to be explored. Previous studies showed that TFRG could inhibit the growth of tumor on nude mice bearing breast cancer [10]. Nevertheless, it remains unknown whether TFRG could inhibit breast cancer by targeting TAMs. So, we evaluated the regulatory effect of TFRG on TAMs* in vitro*.

Macrophages in tumor tissues were mostly polarized to M2 phenotype. The M2-polarized macrophages derived by IL-4, IL-13, IL-10, and glucocorticoids express high levels of Arg-1, CD206 (MRC1), TGF*β*, MMPs, FIZZ1, and YM1, which could promote the metastasis and angiogenesis of tumors [20]. Figure 1 showed that RAW264.7-M2CM attracted 4T1 to elevate its migration, a decreased chemotactic effect of TFRG treated M2-CM to 4T1 breast cells. Figure 2 demonstrated that TFRG could regulate the polarization of macrophages by decreasing the expression of M2-phenotype markers Arg-1 at levels of gene and protein.

Studies have shown that the expression of Arg-1 and iNOS is competitive regulation by Th1 and Th2 cytokines. Arg-1 is induced in response to Th2 cytokines such as IL-4 or IL-13, while iNOS is associated with Th1 cytokines including IFN-*γ* with/not LPS. Arg-1 competes with iNOS for they both can utilize L-arginine as substrate to produce L-ornithine and urea or L-citrulline and NO, respectively [21]. Increased Arg-1 activity reduced availability of L-arginine as the substrate for iNOS in M2 macrophages to limit the antitumor production NO of iNOS. TFRG not only decreased the Arg-1 expression, but also increased the expression of iNOS at the same time (Figures 2 and 3).

The STAT6 signaling pathway is well recognized for its role in the expression of Arg-1 on M2 macrophages induced by IL-4 [22]. By examining the effect of TFRG on the phosphorylation status of STAT6, we found that the level of phosphorylated STAT6 was reduced notably by TFRG (Figure 4(a)). Current studies have revealed a critical role of miR-155 in M2 polarization [23]. Figure 4(b) showed that the level of miR-155 was upregulated by TFRG. We reason that TFRG at least partially acts through increasing the amount of miR-155 in alternatively activated macrophages.

In conclusion, these active extracts and compounds from Qi-tonifying herbs based on M2 macrophage cell model screening may be useful in anticancer therapy by targeting the immunosuppressive activity of TAMs. TFRG is a potential candidate agent for anticancer therapy due to its antitumor effect and immunomodulation, which could inhibit the promoted migration of 4T1 by M2-CM and suppress the polarization of M2, partly via regulating STAT6 signaling pathway and miR-155 expression.

## Figures and Tables

**Table 1:** Ingredients from five herbs.

Pharmacologic name/Chinese name^a^	Produced from	Components
Glycyrrhiza Radix et Rhizoma (Gancao, GC)	root	Glabridin, Isoliquiritin apioside, Liquiritin apioside, Glycyrrhizic acid ammonium salt, Isoliquiritin, Lysionotin
Ginseng Radix et Rhizoma (Renshen, RS)	root	20(S)-Ginsenoside Rg3, Ginsenoside Rg1, Ginsenoside Rf, Lysionotin, Notoginsenoside
Cordyceps (Dongchongxiacao, DCXC)	macrofungi	Cordycepin
Acanthopanacis senticosi Radix et Rhizoma Seu caulis (Ciwujia, CWJ)	root	Eleutheroside B, Hederasaponin B, Eleutheroside E
Astragali Radix (Huangqi, HQ)	root	Astragaloside IV, Calycosin-7-glucoside, Calycosin

a. Based on Chinese Pharmacopoeia (Version 2015).

**Table 2:** The primer of mRNA sequence.

Gene name	Primer sequences
Arg-1	sense 5′-CAGAAGAATGGAAGAGTCAG-3′
	antisense 5′-CAGATATGCAGGGAGTCACC-3′
FIZZ1	sense 5′-CCAACTGTCCTAAGAATGAAGAG-3′
	antisense 5′-AAGCAGGGTAAATGGGCAATA-3′
YM1	sense 5′-GAGTACACAGGCAGGGGTCAATAT-3′
	antisense 5′-GCCAGCAGAAGCTCTCCAGAAG-3′
CD206	sense 5′-GCTGAATCCCAGAAATTCCGC-3′
	antisense 5′-ATCACAGGCATACAGGGTGAC-3′
GAPDH	sense 5′-AG-GTCGGTGTGAACGGATTTG-3′
	antisense 5′-TGTAGACCATGTAGTTGAGGTCA-3′

**Table 3:** Effect of herb extracts and ingredients on Arg-1 mRNA expression and cell viability.

Candidates	Dose^a^	Viability^b^ (%)	Inhibition^c^ (%)
Ethanol extract of GC	100	105.81±4.97	48.21
TFRG	100	107.54±4.98	92.34
Glabridin	50	105.02±2.00	84.38
Isoliquiritin apioside	50	104.05±1.74	53.75
Liquiritin apioside	50	106.79±6.20	41.59
Glycyrrhizic acid ammonium salt	50	94.07±4.36	-
Isoliquiritin	50	97.89±5.25	-
Liquiritin	50	113.24±7.94	-
Ethanol extract of RS	100	92.96±5.52	90.81
Total saponins of RS	100	108.02±7.72	84.30
20(S)-Ginsenoside Rg3	50	95.94±4.30	-
Ginsenoside Rg1	50	97.82±2.08	-
Ginsenoside Rf	50	109.62±5.32	15.06
Lysionotin	50	95.42±2.57	50.98
Notoginsenoside	50	94.08±5.36	-
Ethanol extract of DCXC	100	106.66±2.38	87.09
Cordycepin	50	95.83±1.64	84.40
Ethanol extract of HQ	100	98.86±3.29	65.54
Astragaloside IV	50	106.41±1.86	55.14
Calycosin-7-glucoside	50	98.55±0.97	32.15
Calycosin	50	110.52±6.19	77.51
Ethanol extract of CWJ	100	95.37±8.50	80.65
Eleutheroside B	50	98.97±8.25	30.08
Hederasaponin B	50	98.33±7.20	41.51
Eleutheroside E	50	99.08±1.01	20.52

^**a**^The dosage of herb extracts and fractions was at 100 *μ*g/Ml; the dosage of ingredient was at 50 *μ*M.

^**b**^Cell cytotoxicity: MTT assay was performed to determine cell cytotoxicity of unstimulated RAW264.7 cells treated with herb extracts. Untreated group was considered as 100%.

^**c**^Percent inhibition of Arg-1 expression: qRT-PCR was carried out to measure the production of Arg-1 mRNA in IL-4/IL13-induced RAW264.7 cells in the presence of 100 *μ*g/mL herb extracts and fractions or 50 *μ*M ingredients.

## Data Availability

The data used to support the findings of this study are included within the article.

## Abbreviations

TAMs: Tumor-associated macrophages
TME: Tumor microenvironment
TCM: Traditional Chinese Medicine
qPCR: Quantitative real-time polymerase chain reaction
Arg-1: Arginase-1
FIZZ1: Found in inflammatory zone 1
YM1: Chitinase-3-like protein 3
CD206: Mannose receptor C type 1
iNOS: Inducible nitric oxide synthase
miR-155: miRNA-155
STAT6: Signal transducer and activator of transcription 6
TFRG: Total Flavonoids from Radix Glycyrrhiza.
